# Supplementary material for: Non-linear trends using the generalized additive models in physical and technical performances of the Chinese Super League players
Source: PLoS One. 2024 Jul 26;19(7):e0307843. doi: 10.1371/journal.pone.0307843 (PMC11280146; doi:10.1371/journal.pone.0307843)
Supplement: Attachment — Submitted filename: P1_reviewed.docx [file pone.0307843.s002.docx]

Introduction

1. In line 39, please use current references.
2. In line 42, change Barreira et al
3. In paragraph 2, any reference from the Asian Football Confederation leagues?
4. In line 88, add ‘Chinese Super League (CSL)’

Methods

1. In line 137, does the author analyse the physical and technical indicators of players in different positions?

Discussion

1. Delete line 205-209, Repetitive
2. In the discussion, it will be good to compare your findings with other AFC league teams.
3. In line 229 and throughout the manuscript, change the word ‘more cleanly’ with other appropriate words.
4. Add the limitation of the study and practical implication paragraph.
